# Insights into the mechanism of reversible blood‐brain barrier opening via second near‐infrared region excited gold nanorods photothermal effect: Regulation of the tight junction protein occludin

**DOI:** 10.1002/smo2.70016

**Published:** 2025-08-26

**Authors:** Kaili Liang, Li Yang, Bo Liu, Xinying Wang, Liyan Wang, Jiawei Kang, Zhang Ding, Wei Wang, Qing Wang

**Affiliations:** ^1^ Department of Pharmaceutical Engineering State Key Laboratory of Fine Chemicals School of Chemical Engineering Dalian University of Technology Dalian China; ^2^ Ningbo Institute of Dalian University of Technology Ningbo Zhejiang China

**Keywords:** blood‐brain barrier, occludin, protein conformation, tight junction protein

## Abstract

This study constructed an in vitro blood‐brain barrier (BBB) transwell model to investigate the regulatory effects and mechanisms of the photothermal effects of gold nanorods (AuNRs) excited by the second near‐infrared region (NIR‐II) on BBB permeability. The experimental results showed that the photothermal effects of NIR‐II + AuNRs significantly decreased *trans*‐epithelial electrical resistance (TEER) and increased the permeability of fluorescein isothiocyanate (FITC)‐dextran, indicating that it can effectively open the BBB. This effect was reversible, and the TEER and FITC permeability returned to baseline levels within 24 h after treatment. Mechanistic studies revealed that BBB opening did not rely on apoptosis, cytoskeletal disruption, mitochondrial dysfunction, or inflammation. The opening of the BBB was closely associated with a temporary decrease in the expression and conformational change of the tight junction protein occludin due to the photothermal effect. Molecular simulations and docking analysis revealed that the heat shock protein HSP70 could bind to the conformationally altered occludin, supporting the regulatory role of photothermal effects on tight junction proteins. In summary, NIR‐II + AuNRs achieved safe and reversible opening of the BBB by regulating the conformation and expression of tight junction proteins, providing a deeper insight for further research on BBB and the treatment of neurological diseases.

## Graphical abstract

The photothermal effect generated by second near‐infrared (NIR‐II) laser‐activated gold nanorods (AuNRs) enables reversible blood‐brain barrier (BBB) opening, temporarily increasing its permeability. This process involves the downregulation of tight junction proteins, such as occludin, and triggers their conformational changes.

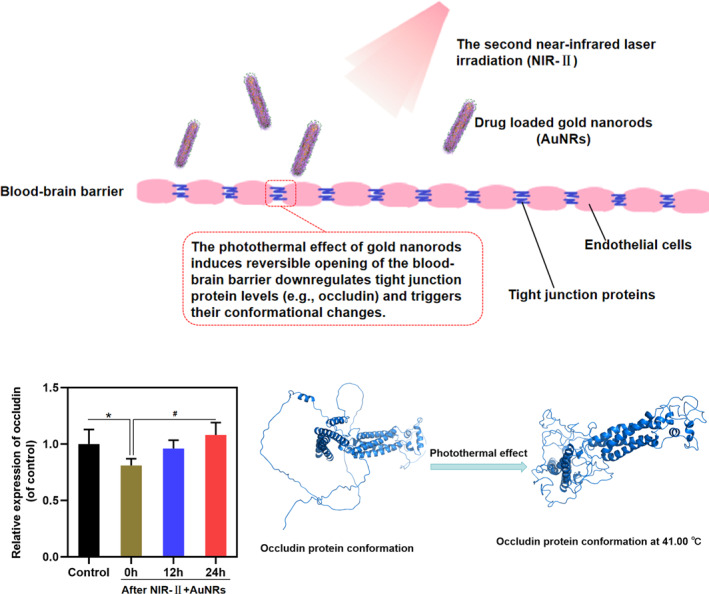

## 1 INTRODUCTION

The blood‐brain barrier (BBB) is a critical barrier that protects the brain against harmful substances in the blood and plays a critical role in maintaining the homeostasis of the central nervous system.[^1^, ^2^, ^3^] Composed of endothelial cells, basement membranes, and glial cells, the BBB maintains its integrity through tight junctions and selective permeability.[^4^, ^5^, ^6^] However, the selective permeability of BBB poses a significant challenge in the delivery of therapeutic drugs and treatment of neurological diseases. Passing through the BBB without causing permanent damage to the brain tissue is a key goal of drug delivery systems.^[7]^ Recently, the combination of nanotechnology and photothermal therapy has provided a new avenue for regulating the BBB.

Gold nanorods (AuNRs) have shown broad application potential in drug delivery and photothermal therapy due to their unique optical properties and tunable surface characteristics. The second near‐infrared region (NIR‐II) light can deeply penetrate tissues, and AuNRs can efficiently convert light energy into thermal energy under NIR‐II irradiation, enabling temperature regulation in local tissues.[^8^, ^9^, ^10^, ^11^] In our previous studies, we employed drug delivery systems based on NIR‐II AuNRs to cross the BBB and deliver levodopa for the treatment of Parkinson's disease.^[12]^ The drug‐loaded nanoparticles were assembled from gold nanorods with a plasmon absorption peak at 1064 nm, poly‐betaine drug carrier polymer, and angiopep‐2 proteins (Figure S1). When laser irradiation was applied to AuNRs, the temperature increased in the system. Real‐time monitoring using an infrared thermography system indicated that the temperature was maintained at 41.00 ± 1.00°C. Our designed smart drug delivery system not only integrates targeted drug delivery and sustained release, but also effectively opens BBB. This method significantly improved the delivery efficiency of levodopa, and enhanced the treatment for Parkinson's disease. This approach of permeabilizing the BBB to enhance drug delivery shows great promise. Building on these findings, we sought to explore the potential mechanisms by which the NIR‐II + AuNRs method permeabilized the BBB.

This study aims to establish an in vitro BBB transwell model to measure the effects of NIR‐II + AuNRs photothermal treatment on the permeability of BBB and explore the mechanism by which this approach permeabilizes the BBB. We expect a deeper understanding of the application potential of NIR‐II + AuNRs in enhancing drug delivery to the brain and provide a theoretical foundation for the development of future therapeutic strategies.

## 2 RESULTS

### 2.1 Construction of in vitro BBB transwell model

**FIGURE 1:**
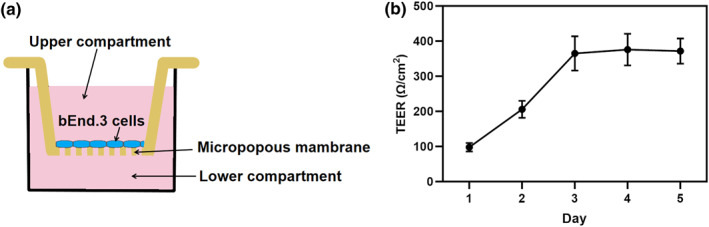
Construction of the BBB model. (a) Overview of the in vitro BBB transwell system. (b) Changes in transendothelial electrical resistance (TEER) values over days during the construction of the BBB transwell model using mouse brain microvascular endothelial cells (bEnd.3). *n* = 3. BBB, blood‐brain barrier.

We constructed an in vitro BBB transwell model to explore the mechanisms by which NIR‐II + AuNRs permeabilize the BBB. The BBB is primarily composed of brain endothelial cells (bEnd.3), which interact with other cell types to maintain its function.[^13^, ^14^] For in vitro BBB Transwell model establishment, bEnd.3 cells are typically employed as the principal component alongside auxiliary cells (e.g., astrocytes) to better recapitulate the complexity of the BBB. In our subsequent experiments, we investigated the therapeutic effects on cytoskeletal dynamics using confocal microscopy. To ensure optimal imaging clarity while minimizing potential interference from co‐cultured cell types, this study specifically employed a bEnd.3 monoculture system to establish the BBB transwell model (Figure 1a). The nanoparticles exhibited no significant toxicity to bEnd.3 cells, as confirmed by the Cell Counting Kit‐8 (CCK‐8) assay (Figure S2). The construction of BBB model was recorded using cell microscopy (Figure S3) and transepithelial electrical resistance (TEER) measurements (Figure 1b). The TEER value was measured as 372 Ω/cm^2^, confirming the successful construction of the BBB model.[^15^, ^16^]

### 2.2 NIR‐II + AuNRs permeabilize the BBB via photothermal effects

**FIGURE 2:**
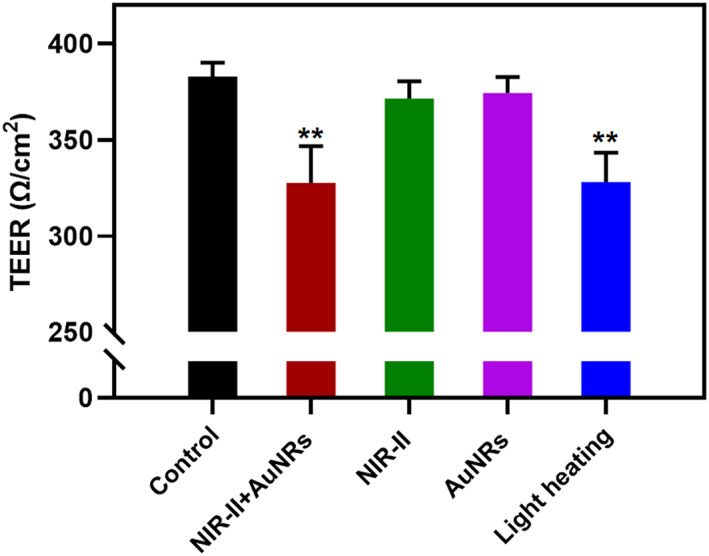
TEER values of the blood‐brain barrier model after exposure to NIR‐II + AuNRs, NIR‐II, or light heating treatment (41.00°C). Compared with the control group (before treatment), **: *P* < 0.01; *n* = 3.

We measured the ability of NIR‐II + AuNRs in permeabilizing the BBB. The experiment was divided into five groups: control, NIR‐II + AuNRs, NIR‐II only, AuNRs only, and heat treatment. The BBB transwell model was treated with different methods, and TEER values were measured after each treatment. The results indicated that after treatment with NIR‐II + AuNRs, the TEER value of the BBB model significantly decreased to 334 Ω·cm^2^ from the baseline value of 378 Ω·cm^2^, indicating that NIR‐II + AuNRs effectively permeabilized the BBB (Figure 2). In our previous study, confocal fluorescence imaging showed that the nanoparticles passed through endothelial cells via endocytosis. However, treatment with AuNRs alone did not lead to a significant decrease in TEER values (Figure 2), suggesting that it failed to open the BBB. This indicates that in the absence of laser irradiation the nanoparticles can cross the BBB through endocytosis without compromising its integrity. When the BBB model was exposed to NIR‐II light or incubated with AuNRs alone, the TEER values did not change significantly compared to the control group, indicating that short‐term NIR‐II irradiation or AuNRs incubation alone could not permeabilize the BBB. However, when the BBB model was heated to 41.00°C for the same duration, the TEER value decreased to 352 Ω·cm^2^, revealing a significant difference compared to the control group. Photothermal treatment with NIR‐II + AuNRs and mild heating could permeabilize the BBB. Therefore, the increased permeability of the BBB after treatment with NIR‐II + AuNRs was due to the increase in temperature induced by the photothermal effect of AuNRs.

### 2.3 NIR‐II + AuNRs reversibly permeabilized the BBB

**FIGURE 3:**
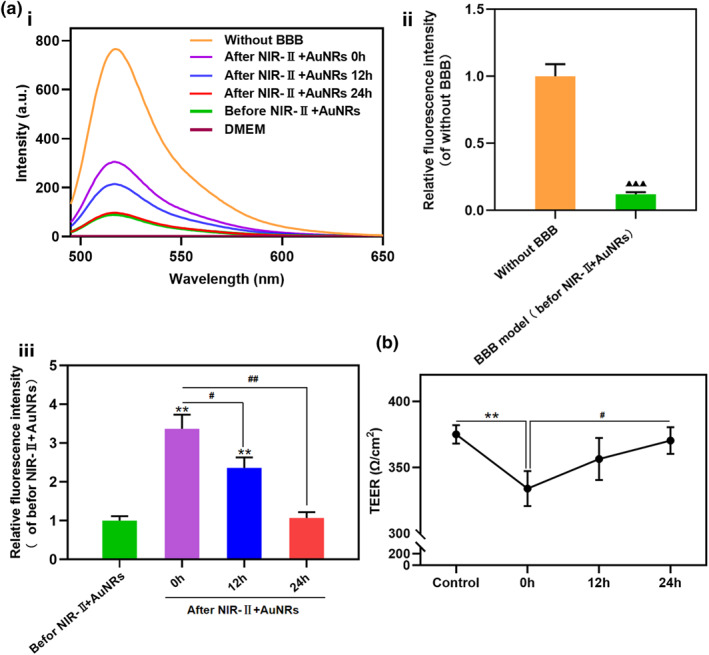
Changes in permeability of the BBB model after different treatments. (a) BBB permeability was assessed with FITC‐dextran. (i) Fluorescence spectra from the lower chamber of the transwell system for each group showing FITC fluorescence. (ii, iii) Comparison of relative fluorescence intensities calculated based on the 530 nm fluorescence peak from panel “i”. (ii) Relative fluorescence intensity in the lower chamber before and after construction of the BBB model used to assess membrane tightness. Compared with the group before BBB construction (without BBB), ▲▲▲: *p* < 0.001. (iii) Relative fluorescence intensity of FITC measured in the lower chamber before and at different time points (0, 12, and 24 h) after NIR‐II + AuNRs treatment, following the addition of FITC‐dextran to the upper chamber. Compared with the group before NIR‐II + AuNRs treatment, **: *p* < 0.01; Compared with the 0 h post‐treatment group, #: *p* < 0.05; ##: *P* < 0.01. (b) TEER values of the BBB model measured before treatment and at different time points (0, 12, and 24 h) after NIR‐II + AuNRs treatment. Compared with the control group (before NIR‐II + AuNRs treatment); **: *p* < 0.01. Compared with the 0 h post‐treatment group, #: *p* < 0.05. *n* = 3. BBB, blood‐brain barrier; FITC, fluorescein isothiocyanate.

Researchers typically analyze BBB permeability using fluorescence labeling methods. Since nanoparticles can cross the BBB via endocytosis, the results evaluating the extent of paracellular pathway opening may be confounded by endocytotic uptake. Therefore, in this study, we employed fluorescein isothiocyanate (FITC)‐dextran to assess BBB opening. As an inert fluorescent macromolecular probe, FITC‐dextran cannot freely traverse an intact tightly confluent endothelial cell layer in in vitro BBB models. It predominantly diffuses passively from the upper chamber to the lower chamber of the Transwell system only when the tight junction structures between cells are disrupted or compromised, leading to increased paracellular permeability. The permeability of FITC‐dextran was measured to determine the extent of BBB opening.[^17^, ^18^] In this study, the BBB transwell model was treated with NIR‐II + AuNRs, and FITC‐dextran was added to the upper chamber at different time points after treatment. The fluorescence intensity of FITC was measured in the lower chamber to evaluate the degree of BBB opening by analyzing the permeability of the FITC‐dextran (Figure 3a). Stronger FITC fluorescence intensity in the lower chamber reflects greater permeation of FITC‐dextran through the BBB, suggesting increased BBB opening. Figure 3ai shows the collected FITC fluorescence spectrum. The relative permeability of FITC‐dextran was determined by comparing the FITC fluorescence intensity at 530 nm, with higher fluorescence intensity indicating greater FITC‐dextran permeability and greater BBB opening. Figure 3aii shows that after establishing the BBB model, the relative fluorescence intensity of FITC dropped to 0.12 times compared to the blank transwell group without a BBB model, indicating a significant decrease in FITC‐dextran permeability. Figure 3aiii shows the relative fluorescence intensity in the lower chamber of the transwell after adding the same dose of FITC‐dextran to the upper chamber at different time points following photothermal treatment. In the group where FITC‐dextran was added to the upper chamber at 0 h post‐treatment, the fluorescence intensity of FITC reached 3.37 times that of the pre‐treatment level. Elevated FITC fluorescence intensity collected from the lower chamber indicated increased translocation of FITC‐dextran from the upper chamber across the BBB into the lower chamber. This demonstrates enhanced FITC‐dextran permeability following photothermal treatment, reflecting greater BBB opening. For the groups where FITC‐dextran was added to the upper chamber at 12 and 24 h post‐treatment, the fluorescence intensities of FITC in the lower chamber were 2.36 times and 1.07 times that of the control, respectively. The progressive decline in the BBB permeation capacity of FITC‐dextran within 24 h suggests that the BBB gradually recovered and NIR‐II + AuNRs‐mediated opening of the BBB was reversible.

Figure 3b shows the changes in TEER before and after treatment with NIR‐II + AuNRs. The results showed that TEER significantly decreased after treatment but returned to baseline within 24 h, confirming that the NIR‐II + AuNRs method reversibly opened the BBB.

### 2.4 BBB opening is independent of cell activity, cytoskeleton, mitochondrial function, or inflammation

**FIGURE 4:**
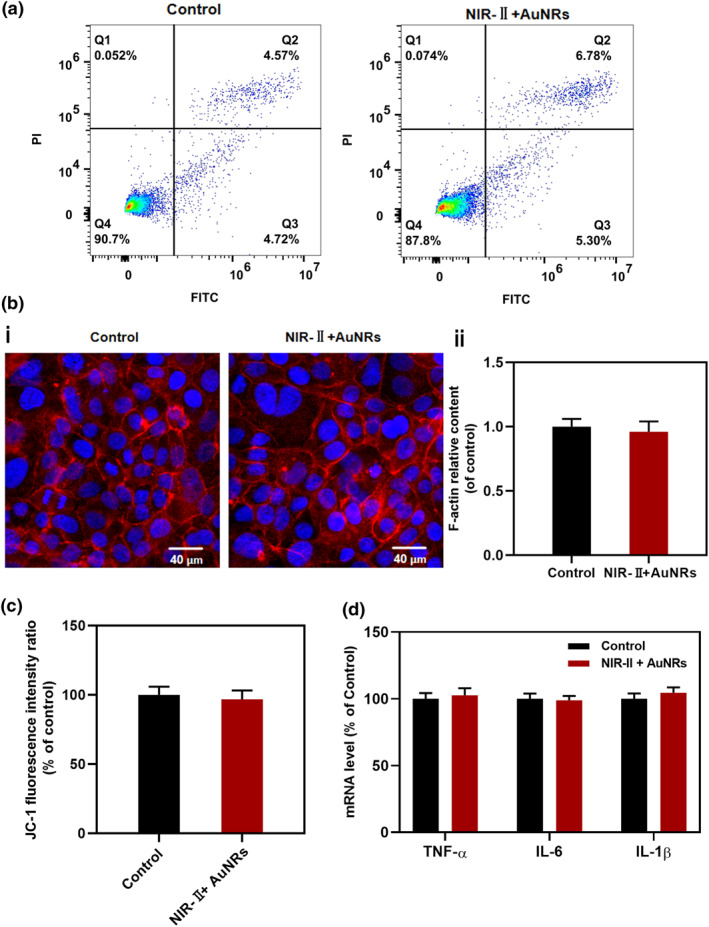
The impact of NIR‐II + AuNRs on the cell activity, cytoskeleton, mitochondrial function, and inflammation. (a) Analysis of cellular apoptosis in the BBB model before and after NIR‐II + AuNRs treatment using flow cytometry. The green fluorescence of Annexin V‐FITC was detected through the FITC channel, and the red fluorescence of PI was detected through the PI channel. (b) Changes in the cytoskeletal protein F‐actin before and after treatment. (i) Fluorescence images of F‐actin stained with phalloidin before and after NIR‐II + AuNRs treatment. Blue: DAPI‐stained nuclei; Red: Alexa Fluor 555‐phalloidin‐stained F‐actin. (Scale bar: 40 μm). (ii) Relative F‐actin content analyzed through fluorescence intensity measurements. (c) Mitochondrial membrane potential of cells before and after treatment. (d) Levels of inflammatory markers (TNF‐α, IL‐6, and IL‐1β) in the BBB model before and after treatment. *n* = 3. BBB, blood‐brain barrier; FITC, fluorescein isothiocyanate; PI, propidium iodide.

Since increased BBB permeability can be affected by endothelial cell apoptosis, cytoskeletal remodeling, cellular metabolism disorders (mitochondrial dysfunction), or inflammatory effects, we investigated whether the opening of the BBB was due to the effects of this method on these aspects. We first analyzed the effects of this method on cell viability.[^19^, ^20^] The results of flow cytometry are shown in Figure 4a. After treatment with NIR‐II + AuNRs, the cells in the BBB model did not show significant apoptosis. We employed phalloidin immunofluorescence staining to label F‐actin and investigated whether the cytoskeleton of the BBB model cells was damaged before and after treatment.[^21^, ^22^] The fluorescence intensity of F‐actin did not show significant changes before and after treatment (Figure 4b), indicating that the increased permeability of the BBB was not due to cytoskeletal damage. Further analysis of mitochondrial membrane potential (Figure 4c) and inflammatory markers, such as TNF‐α, IL‐6, and IL‐1β (Figure 4d), revealed no significant decrease in mitochondrial function or inflammation following treatment, suggesting that NIR‐II + AuNRs did not induce mitochondrial dysfunction or inflammation.

Thus, these experiments confirmed that the NIR‐II + AuNRs method did not open the BBB by inducing cell apoptosis, cytoskeletal damage, mitochondrial dysfunction, or inflammation.

### 2.5 NIR‐II + AuNRs reduced the levels of the tight junction protein occludin

**FIGURE 5:**
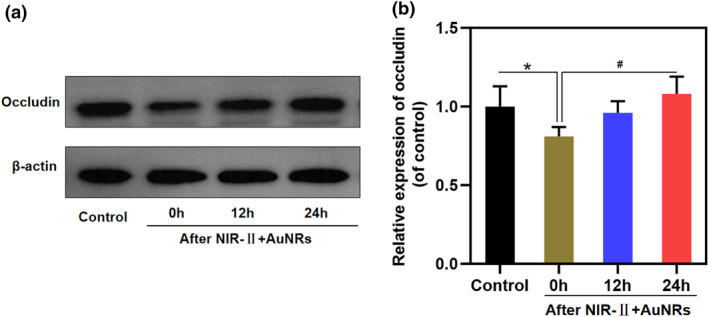
Relative expression of the tight junction protein occludin in the blood‐brain barrier model before and after NIR‐II + AuNRs treatment. (a) Western blot analysis of occludin protein before treatment and at 0, 12, and 24 h after NIR‐II + AuNRs treatment. (b) Occludin relative values before treatment and 0, 12, and 24 h after NIR‐II + AuNRs treatment, calculated based on the densitometry values from panel (a) Compared with the control group (before NIR‐II + AuNRs treatment), *:*p* < 0.05; *n* = 3.

We investigated the effects of NIR‐II + AuNRs on tight junction proteins in the BBB. Occludin is one of the key transmembrane proteins and a key component of cell tight junctions. Together with the claudin family, occludin maintains the selective permeability and structural integrity of the BBB. Its function is not limited to mechanical connections and is involved in signal transduction and dynamic‐barrier regulation. Changes in its content and conformation can affect the tightness of the BBB.[^23^, ^24^, ^25^] We employed occludin protein as a representative to explore whether the NIR‐II + AuNRs method opens the BBB through tight junction proteins. We measured the expression level and conformation of occludin before and after treatment. The occludin level after treatment with NIR‐II + AuNRs was significantly decreased to 0.81 times that of the untreated control group (Figure 5). Therefore, the photothermal effect of NIR‐II + AuNRs downregulated tight junction protein levels in BBB, weakening the tight junctions. The occludin level gradually increased to 0.96 and 1.08 times the control group values after 12 and 24 h of treatment, respectively, indicating that the occludin level gradually recovered after treatment.

### 2.6 NIR‐II + AuNRs induced conformational changes in occludin

**FIGURE 6:**
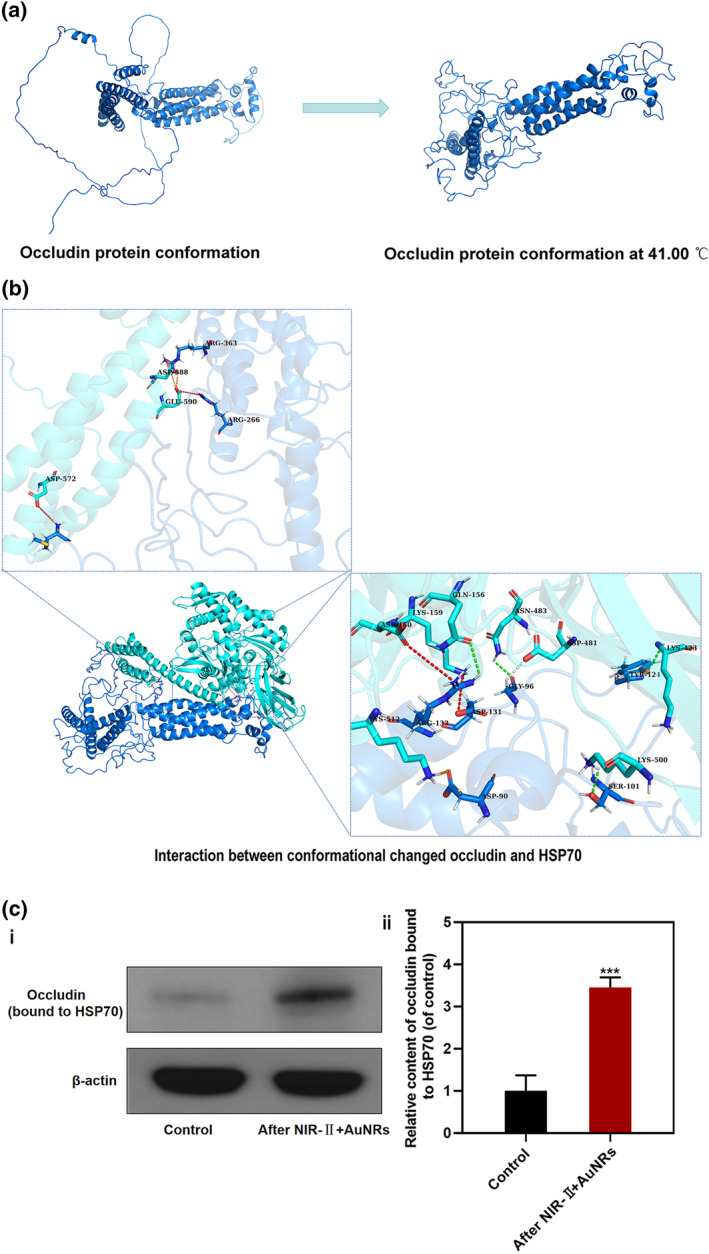
The effect of NIR‐II + AuNRs on the conformation of tight junction protein occludin. (a) Conformational simulation of the occludin protein and its conformation at 41.00°C. (b) Interaction analysis between conformationally changed occludin and HSP70 proteins. (Blue sticks represent occludin protein residues; cyan sticks represent amino acid residues of the HSP70 protein. Green dashed lines indicate hydrogen bond interactions; light green dashed lines represent hydrophobic interactions; orange dashed lines represent salt bridge interactions; red dashed lines denote electrostatic interactions.) (c) Analysis of occludin binding with HSP70. (i) Western blot results showing occludin protein bound to HSP70 in each group after immunoprecipitation (IP), along with WB results of actin in the non‐IP group. (ii) Relative levels of occludin bound to HSP70 before and after NIR‐II + AuNRs treatment, calculated based on the densitometry values from panel (a) ***:*p* < 0.001; *n* = 3.

Next, we investigated whether the photothermal effect of NIR‐II + AuNRs leads to conformational changes in these tight junction proteins. Studies have indicated that the amount and activity of the heat shock protein HSP70 increases under thermal stress, protecting misfolded proteins by preventing aggregation and assisting their correct refolding after returning to normal conditions.[^26^, ^27^] We also detected a significant increase in HSP70 activity after photothermal treatment (Figure S4).^[28]^ Therefore, HSP70 can be used to detect protein unfolding after thermal stress. We first used molecular simulations to measure the conformational changes in occludin after exposure to the photothermal effect and measure its interaction with HSP70. Since the temperature of 41.00°C can be used for therapeutic purposes in biological systems[^29^, ^30^] and during treatment with NIR‐II + AuNRs, we controlled a temperature of 41.00 ± 1.00°C to permeabilize the BBB. We conducted dynamic simulations of occludin at 41.00°C, and found that the conformation of occludin changed at this temperature (Figure 6a). We performed molecular docking to study the nature of their interaction to explore whether HSP70 interacts with occludin after heat‐induced conformational change (Figure 6b, Figure S5 and Table S1). The docking was performed using the HDCOK program, which is specialized for protein‐protein and protein‐DNA/RNA interactions. This was a global docking analysis, and the best‐scoring structure was selected for further interaction analyses. The results of molecular docking are shown in Figure 5b and Table S1, with a confidence score of 0.99, significantly higher than the 0.70 threshold.[^31^, ^32^, ^33^] This finding indicates that the docking complex model is highly reliable. Molecular docking showed high binding affinity between conformational changes of occludin protein and HSP70.

Next, we validated the binding of conformationally changed occludin with HSP70. We extracted HSP70 and its protein complex and detected the content of occludin bound to HSP70 before and after treatment. The results are shown in Figure 6c. After treatment with NIR‐II + AuNRs, the amount of occludin bound to HSP70 was 3.45 times that before treatment, confirming the binding of occludin to HSP70 after the photothermal effect. This is consistent with the role of HSP70 as a molecular chaperone binding to unfolded proteins.

These findings suggest that occludin undergo conformational changes after treatment with NIR‐II + AuNRs.

## 3 DISCUSSION

This study systematically investigated the regulatory effects and mechanisms of the photothermal effects of NIR‐II + AuNRs on BBB permeability using an in vitro transwell model of BBB. The results demonstrated that NIR‐II + AuNRs induce reversible BBB opening through mild photothermal effects (41.00°C), characterized by conformational changes and transient downregulation of the tight junction protein occludin. Notably, this process is independent of apoptosis, cytoskeletal disruption, mitochondrial dysfunction, or inflammatory responses.

Our study first validated the efficacy of the NIR‐II + AuNRs complex in opening the BBB. Measurements of TEER (Figure 2) revealed a significant decrease in TEER in the NIR‐II + AuNRs treatment group, suggesting BBB opening. Importantly, neither NIR‐II irradiation alone nor AuNRs incubation alone altered TEER values. On the contrary, the photothermal effect of NIR‐II‐activated AuNRs effectively reduced TEER, confirming that localized increase in temperature induced by NIR‐II + AuNRs is the key mechanism modulating BBB.

Further assessment indicated that the permeabilization of the BBB induced by photothermal effects was reversible (Figure 3). FITC‐dextran permeability significantly increased after treatment but returned to baseline levels within 24 h. Dynamic TEER recovery was also consistent with the transient nature of BBB opening, highlighting its clinical relevance. Such reversible modulation minimizes long‐term cerebral effects while enhancing drug delivery safety.

We analyzed the effects of this method on cell viability, cytoskeleton, mitochondrial function, and inflammatory responses to explore the potential mechanisms by which NIR‐II + AuNRs permeabilize the BBB (Figure 4). The results showed that treatment with NIR‐II + AuNRs did not induce apoptosis, cytoskeletal damage, mitochondrial dysfunction, or inflammation, excluding the role of these pathways in BBB permeability. The BBB‐opening approach employed in this study does not induce inflammation, which distinguishes it from other stimulation‐based methods. For instance, while focused ultrasound can effectively induce BBB opening (with recovery occurring 6–72 h after treatment), this method elicits significant inflammatory responses. The inflammatory reaction peaks at 24 h and persists until complete resolution at 72 h after treatment.[^34^, ^35^] In contrast, the NIR‐II + AuNRs method achieves BBB opening without inducing significant inflammatory reactions. Furthermore, the photothermal effect of NIR‐II + AuNRs did not lead to significant cellular damage or metabolic abnormalities, providing complete recovery of cellular function in BBB.[^36^, ^37^, ^38^] In contrast, treatment with high‐temperatures (such as 60.00°C) induced cell damage and mitochondrial dysfunction (Figure S6), which impaired cell recovery and BBB restoration within 24 h.

Further studies revealed that photothermal effects regulate BBB permeability by modulating tight junction proteins.[^39^, ^40^] We found that the expression level of occludin significantly decreased and its conformation changed after treatment with NIR‐II + AuNRs. Occludin is one of the key proteins that maintain tight junctions in the BBB, and changes in its level (Figure 5) and conformation can lead to the relaxation of tight junctions (Figure 6), thereby increasing the permeability of BBB. Molecular simulation and docking analysis revealed significant conformational changes in occludin after treatment with NIR‐II + AuNRs. This change may lead to its binding to HSP70. HSP70 binding supports the conformational unfolding of occludin. HSP70, as a molecular chaperone, can help proteins affected by thermal stress recover their function or folding state, supporting photothermal effect‐mediated modulation of tight junction proteins.[^27^, ^41^] In contrast, the binding of occludin to HSP70 was notably impaired after 60°C (Figure S7). The amount of occludin bound to HSP70 after 60°C was only 2.08 times that of the pre‐treatment level, significantly lower than the 3.45‐fold increase observed under mild hyperthermia. This suggests that while HSP70 retains partial binding affinity for denatured proteins at 60°C, its protective capacity is substantially compromised compared with milder thermal conditions. This limitation could be attributed to several factors. On one hand, the 60°C treatment exceeds the physiological temperature threshold (with protein denaturation starting at >45°C), causing more extensive damage to the proteins, which may lead to irreversible aggregation of tight junction proteins and partial inactivation of HSP70, which has a denaturation temperature of nearly 60–65°C. On the other hand, treatment at 60°C induces cellular damage and mitochondrial dysfunction. Severe cellular damage interferes with protein recovery. Since HSP70 protects proteins through an ATP‐dependent cycle, impairment of mitochondrial function hinders the cell's ability to supply the energy needed for this protective process.^[42]^ In this study, photothermal effects induced a reduction in occludin protein levels. Consistent with this, compared with pre‐treatment levels, the protein levels of tight junction proteins ZO‐1 and claudin‐5 showed a significant decrease after treatment and recovered within 24 h; no significant change was observed in the level of adhesion junction protein VE‐cadherin (Figure S8). Therefore, NIR‐II + AuNRs facilitate the opening of the BBB by modulating tight junction proteins.Notably, treatment with NIR‐II + AuNRs may decrease tight junction protein levels due to accelerated degradation through the proteasomal or lysosomal pathways,[^43^, ^44^, ^45^] but its recovery to baseline levels within 24 h suggests that the photothermal effects did not irreversibly inhibit protein synthesis. This dynamic balance may be regulated via cellular stress response pathways,[^46^, ^47^] such as heat shock factor 1 (HSF1), although further studies are needed to elucidate the exact mechanisms.

## 4 CONCLUSION

In summary, this study systematically investigated the regulatory effects of the photothermal effect of NIR‐II + AuNRs on the permeability of the BBB using an in vitro BBB transwell model. Our findings demonstrated that NIR‐II + AuNRs can reversibly permeabilize the BBB through photothermal effects, primarily by affecting the expression and conformation of the tight junction protein occludin, rather than by inducing apoptosis, cytoskeletal damage, mitochondrial dysfunction, or inflammatory responses. This study advances our understanding of BBB regulation and offers a promising strategy for targeted drug delivery in brain disorders.

## 5 MATERIALS AND METHODS

### 5.1 Materials

Fetal bovine serum (FBS) was purchased from Tianhang Biotechnology Co., Ltd.. Dulbecco's Modified Eagle's Medium‐High Glucose (DMEM‐H) was obtained from Solarbio Science & Technology Co., Ltd. (Beijing, China). Fluorescein isothiocyanate (FITC)‐Dextran (20 kDa) was purchased from Titan Scientific Co., Ltd.. Mitochondrial Membrane Potential Assay Kit with JC‐1 was purchased from Solarbio (Beijing, China).The Annexin V‐FITC/Propidium Iodide (PI) Apoptosis Detection Kit, the Cell Counting Kit‐8 (CCK‐8) for cell proliferation and cytotoxicity assays, enzyme‐linked immunosorbent assay (ELISA) kit, 4′,6‐diamidino‐2‐phenylindole (DAPI) nuclear stain, occludin mouse monoclonal antibody, β‐actin mouse antibody, Alexa Fluor 555‐conjugated phalloidin, enhanced chemiluminescence (ECL) reagent, and Radio Immunoprecipitation Assay (RIPA) lysis buffer were obtained from Servicebio Technology Co., Ltd.. The remaining compounds were all analytical grade and used without further modification.

### 5.2 Cell culture

The immortalized mouse brain microvascular endothelial cell line (bEnd.3) was obtained from Beina Biotechnology Research Institute (Beijing, China). Cells were cultured in DMEM‐H supplemented with 10% FBS at 37°C in an incubator with 5% CO_2_.

### 5.3 In vitro BBB transwell model construction and photothermal treatment

In vitro BBB transwell model was constructed using bEnd.3 cells. The cells (1 × 10^5^ cells/cm^2^) were seeded into the upper compartment of a transwell insert and cultured in DMEM‐H containing 10% FBS. During the culture period, transepithelial electrical resistance (TEER) was measured daily. TEER was measured using a Millicell ERS epithelial voltmeter (Millipore).

To investigate changes in BBB permeability induced by the photothermal effect of NIR‐II irradiation, nanoparticles were added to the culture medium, and the samples were irradiated with a 1064 nm NIR laser (0.80 W/cm^2^, 41.00 ± 1.00°C, 10 min), and the temperature was monitored through an infrared imaging system. Throughout this period, irradiation was paused when the temperature exceeded 42.00°C and resumed when it decreased to 40.00°C, maintaining the therapeutic temperature at 41.00°C ± 1.00°C. As demonstrated in Figure S9B, the temperature profile of the system under continuous laser irradiation at 0.8 W/cm^2^ confirms that the maximum achievable temperature exceeded 41.00°C, thus fulfilling the requirement for our therapeutic temperature range.

The integrity of the model was assessed by TEER measurements both before and after treatment.

### 5.4 FITC‐dextran BBB permeability assay

After the cells form a dense monolayer, conduct permeability testing experiments on the model. A 0.1 mL solution of FITC‐dextran (0.5 mg/mL in serum‐free, phenol red‐free DMEM) was added to the upper chamber of the transwell, and 0.6 mL of DMEM without FITC‐dextran was added to the lower chamber. Samples were taken out of the transwell device's upper and bottom chambers for the purpose of detecting fluorescence intensity after an hour of incubation in a cell culture incubator.

### 5.5 Flow cytometry protocol

The BBB model cells were digested and washed, and then resuspended in 100 μ L of 1 × binding buffer.The cell pellet was then resuspended in 100 μL of 1 × Binding Buffer. Subsequently, 5 μL of Annexin V‐FITC was added and mixed thoroughly, followed by the addition of 5 μL of PI with gentle mixing. The samples were incubated at room temperature in the dark for 15 min. After staining, 400 μL of 1 × Binding Buffer was added to each tube, and the samples were analyzed using a flow cytometer.

The green fluorescence of Annexin V‐FITC was detected through the FITC channel (FL1), and the red fluorescence of PI was detected through the PI channel (FL2). The flow cytometer parameters were as follows: excitation wavelength (Ex) = 488 nm, emission wavelength for FL1 (Em = 525 ± 20 nm), and for FL2 (Em = 585 ± 21 nm). The proportions of cells in different states were analyzed using FlowJo software.

### 5.6 Mitochondrial membrane potential assay

Mitochondrial membrane potential was assessed using the JC‐1 Mitochondrial Membrane Potential Assay Kit. Cells were washed with PBS and incubated with 1 mL of JC‐1 staining working solution at 37°C for 20 min in a cell culture incubator. Then, the cells were washed twice with JC‐1 staining buffer (1×), followed by the addition of fresh cell culture medium. Fluorescence intensity was measured using a microplate reader with excitation and emission wavelengths set to Ex = 488 nm and Em = 590 nm, respectively.

### 5.7 Inflammatory analysis

Expression of inflammatory cytokine mRNA was analyzed by quantitative polymerase chain reaction (qPCR). Total RNA was extracted by lysing PBS‐washed cells in RNA lysis buffer, followed by centrifugation (12,000 rpm, 10 min, 4°C). The supernatant was mixed with RNA Extraction Buffer, centrifuged, and combined with isopropanol. After −20°C incubation (15 min), RNA pellets were washed with 75% ethanol, centrifuged (12,000 rpm, 5 min, 4°C), and dissolved in RNase‐free water (60°C, 10 min).

Reverse transcription was performed using a reverse transcription kit. Reverse transcription used a 20 μL mix containing 5 × buffer, oligo(dT)/random hexamer primers, reverse transcriptase, RNA, and water. Reactions proceeded in a thermal cyclerat 25°C (5 min), 42°C (30 min), 85°C (5 s).

Then, qPCR reactions (0.1 mL plates) included 2 × SYBR Green Master Mix, primers, cDNA, and water. Plates were sealed, centrifuged, and amplified in a real‐time PCR system (ETC811, Servicebio, Wuhan, China). Cycling conditionswere 95°C (30 s), 40 cycles (95°C/15 s, 60°C/30 s), and melt curve analysis (65–95°C). Relative mRNA levels were quantified and normalized to housekeeping genes.

### 5.8 Phalloidin immunofluorescence staining

The cells were dried, circled with a histochemical pen, and permeabilized (100 μL permeabilization solution, 20 min). After three PBS washes (5 min each), the Alexa Fluor 555‐phalloidin solution was added and incubatee for two hours in the dark. The cells were then stained with DAPI (10 min). Images were captured using a laser scanning confocal microscope (Olympus, Japan) and further processed with AIpathwell software (Servicebio Technology Co., Ltd.).

### 5.9 Analysis of occludin protein expression

The expression of occludin was detected through Western blot (WB) method. The proteins were extracted using RIPA buffer, denatured in 5 × sample buffer (4:1) and boiled (15 min). The protein samples were subjected to sodium dodecyl sulfate‐polyacrylamide gel electrophoresis (SDS‐PAGE), and then transferred to activated polyvinylidene fluoride (PVDF) membranes. The PVDF membrane was blocked with 5% nonfat milk for 1 h and then incubated with the primary antibody overnight at 4°C. After three quick washes, the membrane was treated with HRP‐conjugated secondary antibody (1 h). After ECL development, band intensity was quantified using ImageJ software.

### 5.10 Molecular dynamics simulation of occludin

To determine the protein conformation of occludin at 41.00°C, a 100 ns molecular dynamics simulation of the occludin protein was performed at 314.15 K (41.00°C) and 1 bar pressure (GROMACS 2021 software). Newton's equations of motion were integrated using the leapfrog algorithm with a time step of 2 fs. Temperature coupling was applied using the V‐rescale method, and pressure coupling was handled with the Parrinello‐Rahman method. Neighbor searching was performed using the Verlet algorithm. Long‐range electrostatic interactions were treated using the PME method. Long‐range dispersion corrections were applied to energy and pressure calculations.

To investigate the binding regions and interaction modes between Occludin and HSP70, a professional protein‐protein and protein‐DNA/RNA docking program HDCOK was used. The docking performed was global docking. After docking, the structure with the best docking score was selected as the reference for further interaction analysis. The docking score was calculated based on the ITScorePP or ITScorePR iterative scoring functions. The confidence score of the docking, which indicates the likelihood of binding between the two molecules, was calculated as follows:

Confidencescore=1.0/1.0+e^(0.02∗(dockingscore+150))

### 5.11 Analysis of HSP70‐occludin interaction

Firstly, the immunoprecipitation method was used to extract HSP70 and the protein conjugates that bind to HSP70. Briefly, the extracted protein sample was incubated with a pre‐prepared HSP70 antibody‐SweMagrose Protein G magnetic bead working solution at 4°C overnight. After incubation, the sample was placed on a magnetic separator for 10 s to remove the supernatant, followed by three washes with 1 × TBS. Next, 1 × protein loading buffer (reduced form) was added to the pellet, mixed thoroughly, and heated at 95°C for 5 min to denature the proteins and disrupt the interactions between proteins and between proteins and magnetic beads. Magnetic separation was then performed, and the supernatant was collected. The occludin protein content in the supernatant was analyzed using the WB method.

### 5.12 Statistical analysis

When applicable, one‐way or two‐way analysis of variance (ANOVA) is used to examine the data, which are displayed as mean ± standard deviation. A significance threshold of *p* < 0.05 was considered statistically significant.

## CONFLICT OF INTEREST STATEMENT

The authors declare no conflicts of interest.

## ETHICS STATEMENT

All experiments were conducted following the guidelines of Dalian University of Technology for the welfare of experimental animals (Approval number: DUTSCE220813_01).

## Supporting information

Supporting Information S1

## Data Availability

All data generated or analyzed during this study are included in this published article.
