# Supplementary material for: Insights into the mechanism of reversible blood‐brain barrier opening via second near‐infrared region excited gold nanorods photothermal effect: Regulation of the tight junction protein occludin
Source: Smart Mol. 2025 Aug 26;3(3):e70016. doi: 10.1002/smo2.70016 (PMC12483132; doi:10.1002/smo2.70016)
Supplement: Supplementary file 1 — Supporting Information S1 [file SMO2-3-e70016-s001.docx]

Insights into the mechanism of reversible blood-brain barrier opening via NIR-II excited gold nanorods photothermal effect: regulation of the tight junction protein occludin

Kaili Liang^1^, Li Yang^1,2^, Bo Liu^1^, Xinying Wang^1^, Liyan Wang^1^, Jiawei Kang^1^, Zhang Ding^1^, Wei Wang^1^, Qing Wang^1^*

*^1^*State Key Laboratory of Fine Chemicals, Department of Pharmaceutical Engineering, School of Chemical Engineering, Dalian University of Technology, No. 2, Linggong Road, Ganjingzi District, Dalian 116024, China

Email: qwang@dlut.edu.cn (Q. Wang)

*^2^*Ningbo Institute of Dalian University of Technology, No.26, Yucai Road, Jiangbei District,

Ningbo, Zhejiang 315016, China


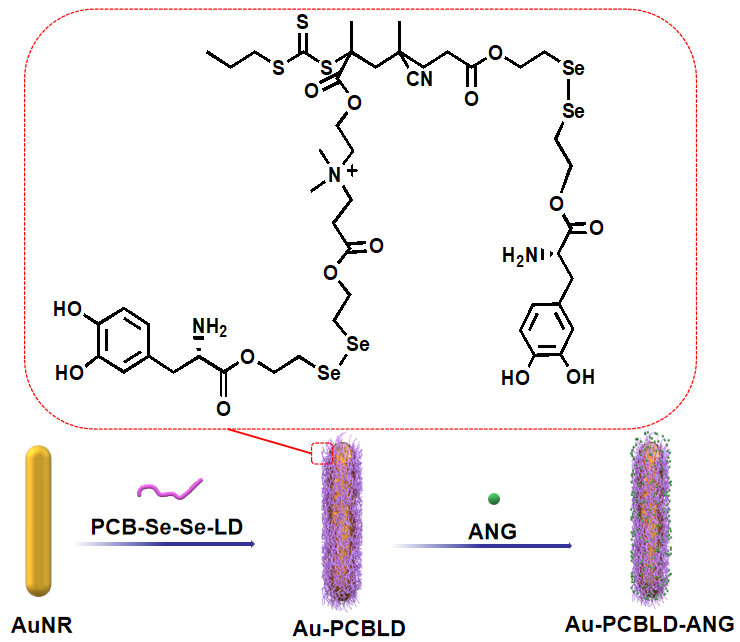


**Figure S1.** Composition and structure of drug loaded gold nanorods (AuNRs)^[1]^. The drug delivery principle of this smart drug delivery system is as follows: (1) This strategy synergistically enhances drug delivery through dual mechanisms: targeted blood-brain barrier (BBB) accumulation of AuNRs via ligand-receptor interactions, followed by receptor-mediated endocytosis for brain entry; (2) photothermal BBB modulation using gold nanorods, where the second near-infrared (NIR-Ⅱ) irradiation triggers localized hyperthermia to transiently disrupt the BBB, amplifying nanoparticle penetration.

Post-brain entry, the polymeric nanoparticles leverage their diselenide bond-containing architecture to execute dual therapeutic actions: (1) reactive oxygen species (ROS)-responsive drug release: The diselenide bonds undergo selective cleavage in the elevated ROS microenvironment of Parkinsonian brains, enabling sustained, site-specific levodopa delivery. (2) ROS scavenging: neutralizing pathological ROS levels, ameliorating oxidative stress and restoring the cerebral microenvironment.


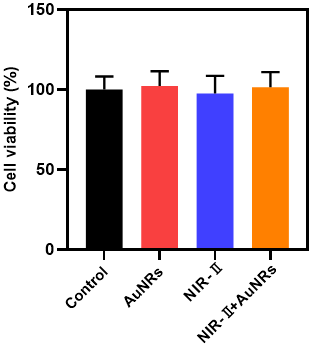


**Figure S2.** Cytotoxicity of drug loaded gold nanorods (AuNRs), second near-infrared (NIR-II) light , and NIR-II+ AuNRs groups. Cell viability was assessed using the cell counting kit-8 (CCK-8) assay to evaluate cytotoxicity, n=3.


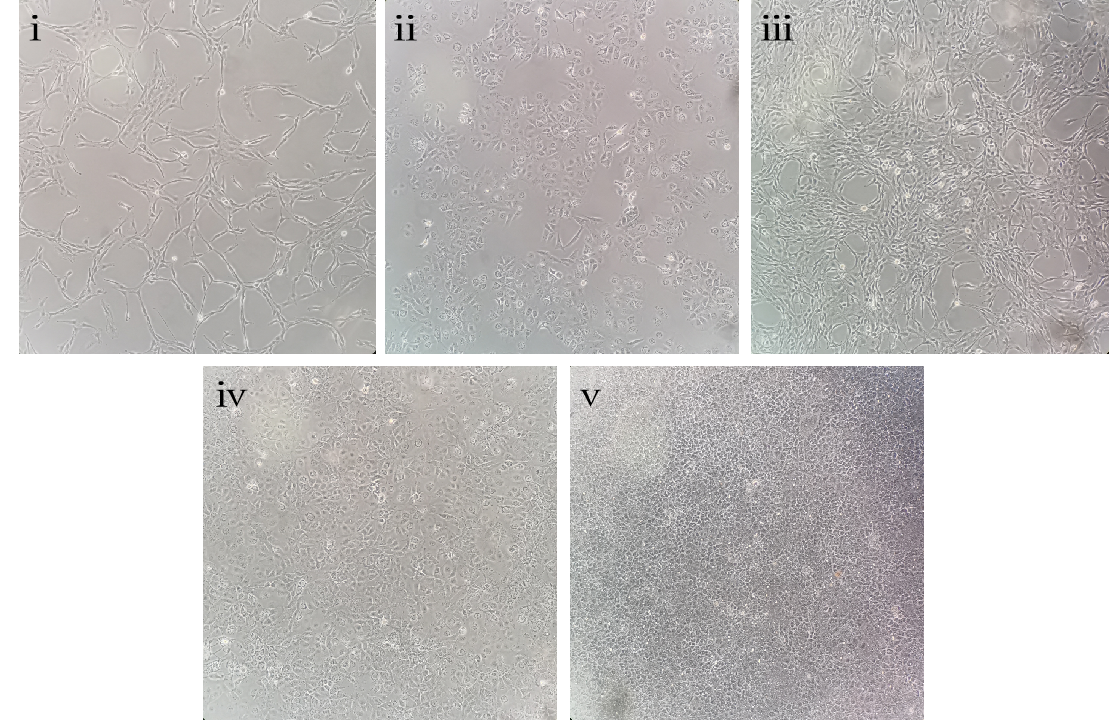


**Figure S3.** The formation process of the blood-brain barrier (BBB) model captured via cellular microscopy (days 1 to 5).


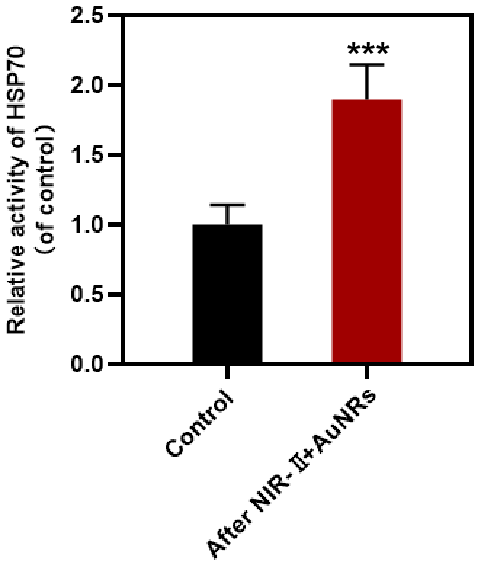


**Figure S4.** Relative activity of HSP70 before and after NIR-II+ AuNRs treatment, measured by ELISA. Compared to the control group, ＊＊＊: P < 0.001, n=3.


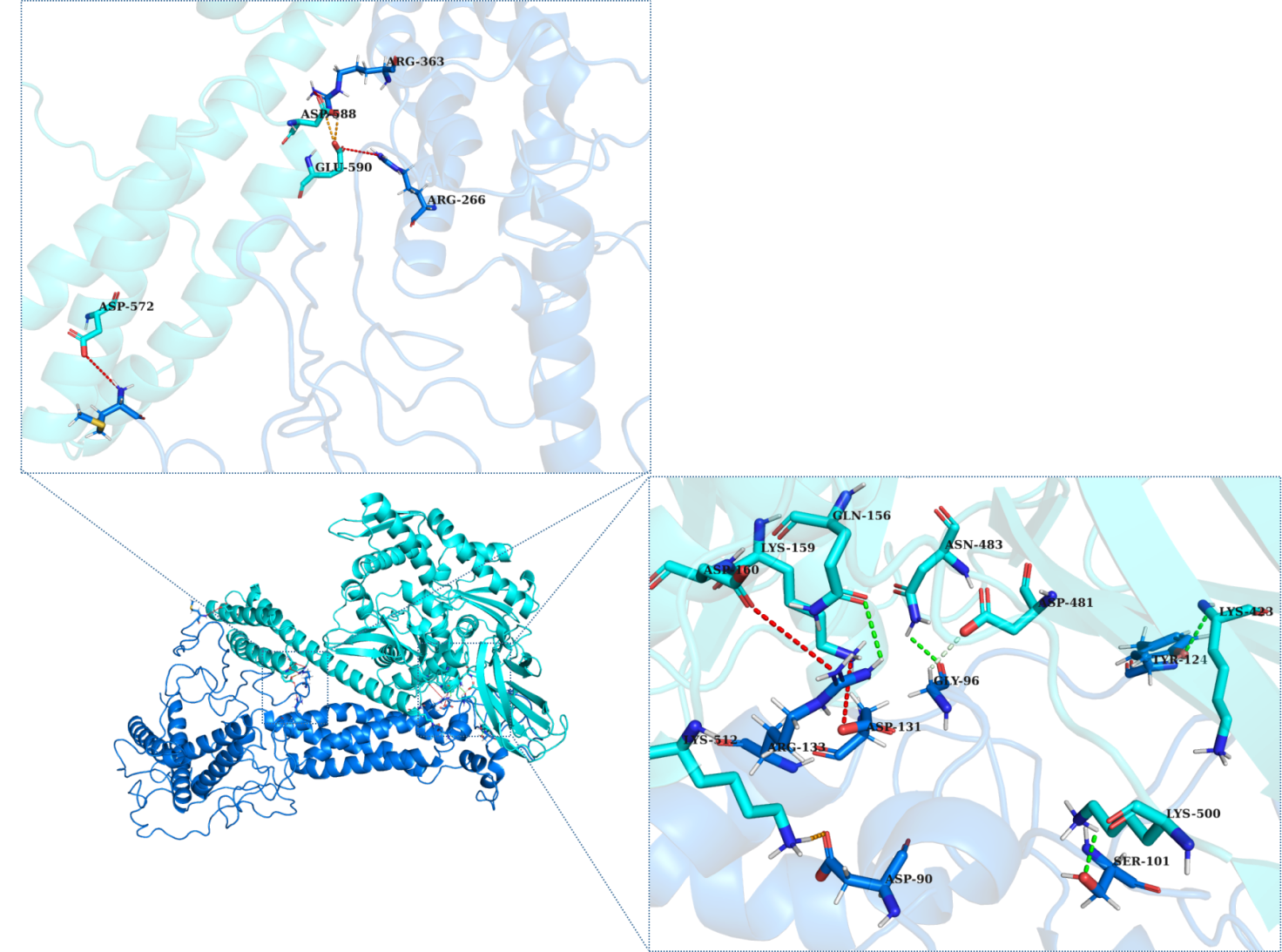


**Figure S5.** Interaction analysis between conformationally changed occludin and HSP70 proteins. Blue sticks represent occludin protein residues; cyan sticks represent amino acid residues of the HSP70 protein. Green dashed lines indicate hydrogen bond interactions; light green dashed lines represent hydrophobic interactions; orange dashed lines represent salt bridge interactions; red dashed lines denote electrostatic interactions.


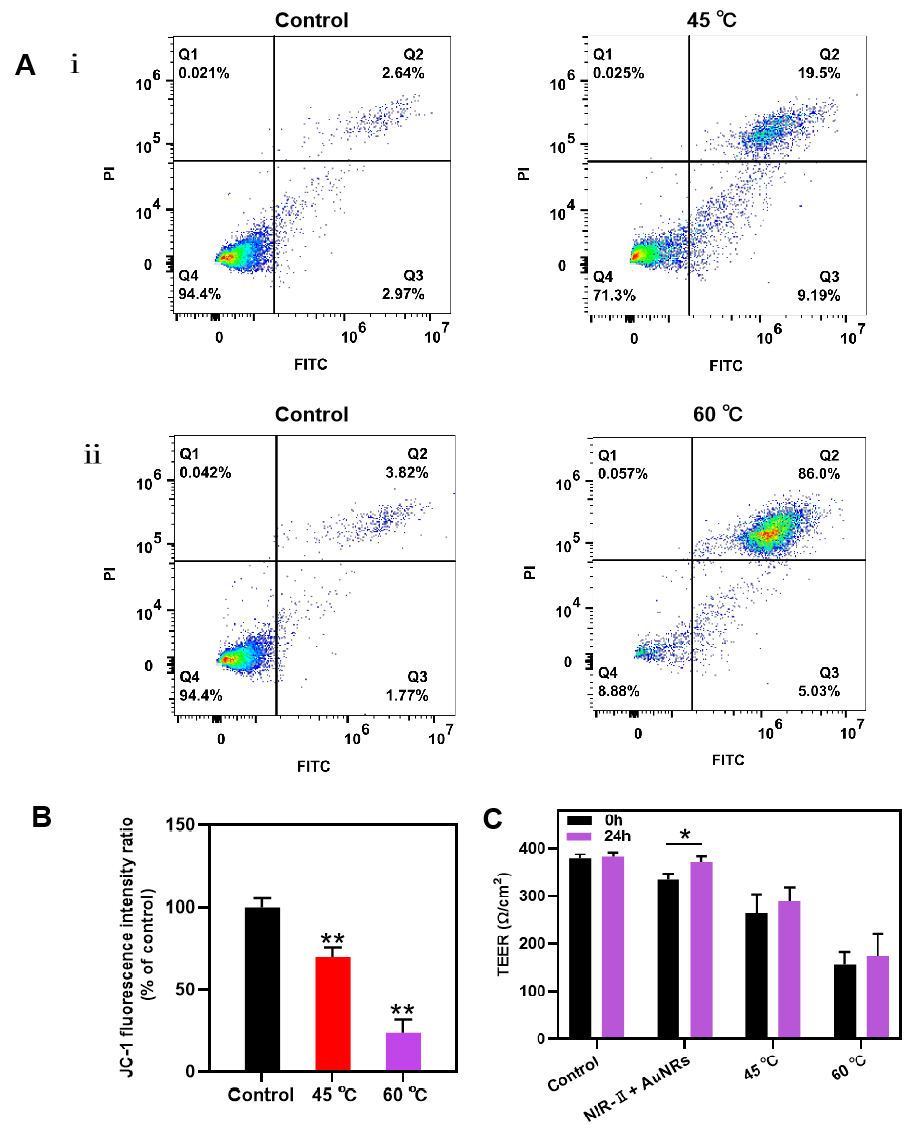


**Figure S6.** Impact of 45°C and 60°C on the bEnd.3 cell-based BBB model. (A) Apoptotic response of bEnd.3 cells following 45 °C and 60 °C thermal exposure analyzed by flow cytometry. The green fluorescence of Annexin V-Fluorescein isothiocyanate (FITC) was detected through the FITC channel, and the red fluorescence of Propidium Iodide (PI) was detected through the PI channel. (B) Mitochondrial membrane potential in BBB model cells under 45 °C and 60 °C treatment. Compared to the control group, ＊＊: P < 0.01, n=3. (C) Impact of 45 °C and 60 °C stress on transendothelial electrical resistance (TEER) in the BBB model. Compared to the 0 hs after treatment, ＊: P < 0.05, n=3.


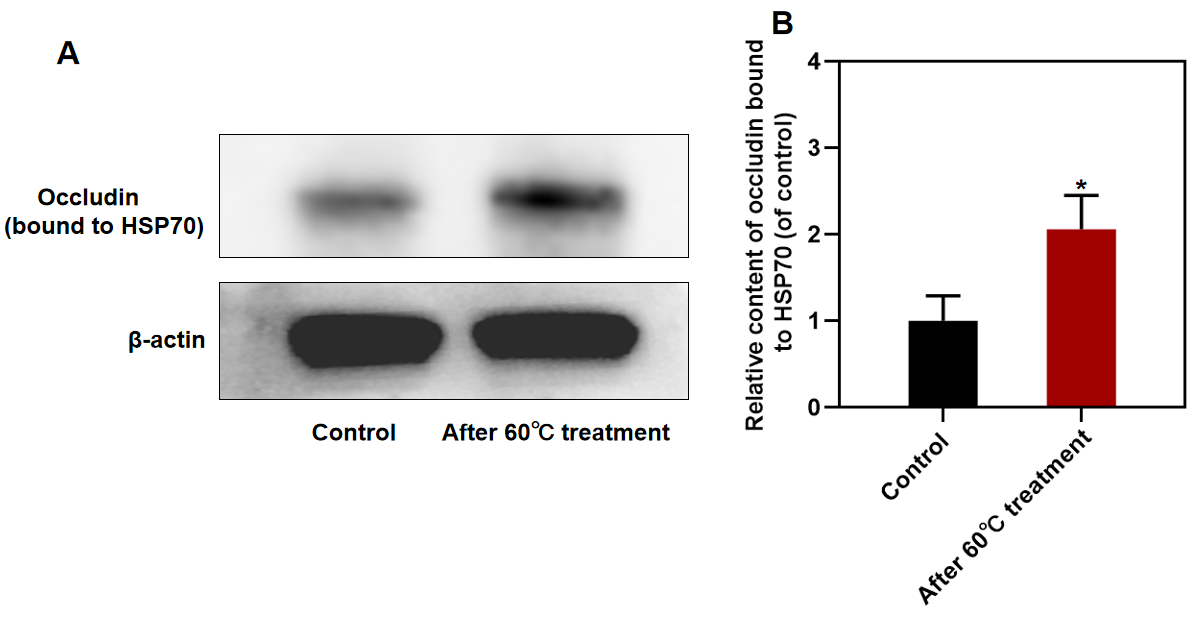


**Figure S7** Analysis of occludin binding with HSP70 after 60℃ treatment. (A) WB results showing occludin protein bound to HSP70 in each group after immunoprecipitation (IP), along with WB results of actin in the non-IP group. (B) Relative levels of occludin bound to HSP70 before and after 60 ℃ treatment, calculated based on the densitometry values from panel A. Compared to the control group, ＊: P < 0.05, n=3.


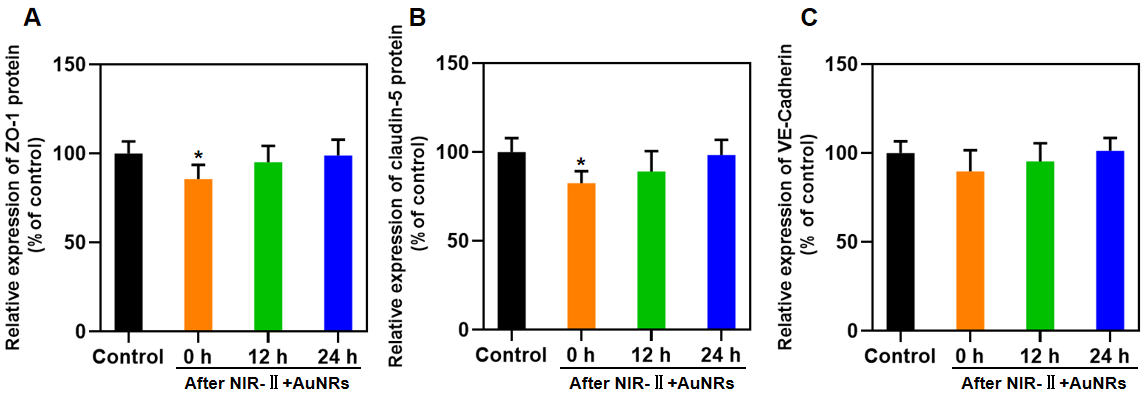


**Figure S8** Quantification of (A) ZO-1, (B) claudin-5, and (C) VE-cadherin levels by ELISA before and at different time points after NIR-II+AuNR treatment. Compared to the control group, ＊: P < 0.05; n = 3.


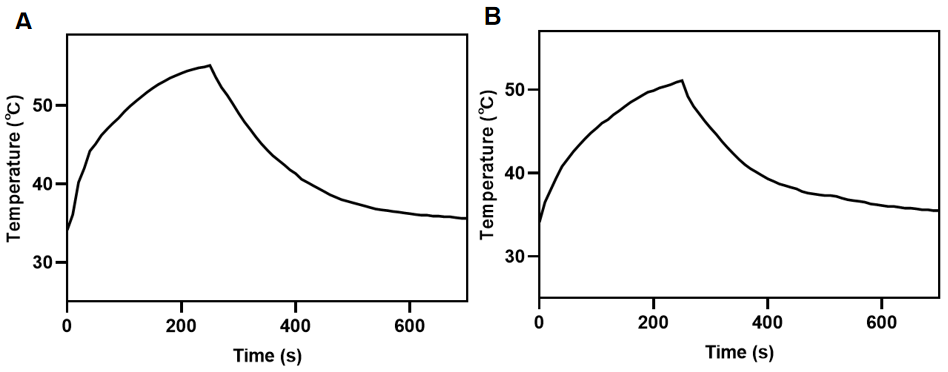


**Figure S9** Heating and cooling curves of the system under 1064 nm laser irradiation. (A) The temperature variations were monitored over the heating and cooling cycles under 1 W/cm² laser power density; (B) The temperature variations were monitored over the heating and cooling cycles under 0.8 W/cm² laser power density.

**Table S1.** Amino acid residues involved in the interaction between occludin and HSP70.

| Name | Category |
| --- | --- |
| Occludin:ARG363:HH21 - HSP70:GLU590:OE2 | Salt Bridge |
| Occludin:ARG363:HH22 - HSP70:GLU590:OE2 | Salt Bridge |
| HSP70:LYS512:HZ1 - Occludin:ASP90:OD1 | Salt Bridge |
| Occludin:MET1:N - HSP70:ASP572:OD2 | Electrostatic |
| Occludin:ARG133:NH1 - HSP70:ASP160:OD1 | Electrostatic |
| Occludin:ARG266:NH2 - HSP70:GLU590:OE1 | Electrostatic |
| Occludin:ARG363:NH1 - HSP70:ASP588:OD1 | Electrostatic |
| Occludin:ARG363:NH2 - HSP70:ASP588:OD2 | Electrostatic |
| HSP70:LYS159:NZ - Occludin:ASP131:OD2 | Electrostatic |
| Occludin:ARG133:HH22 - HSP70:GLN156:OE1 | Hydrogen Bond |
| HSP70:LYS423:HN - Occludin:TYR124:OH | Hydrogen Bond |
| HSP70:ASN483:HD22 - Occludin:GLY96:O | Hydrogen Bond |
| HSP70:LYS500:HZ3 - Occludin:SER101:OG | Hydrogen Bond |
| Occludin:GLY96:HA1 - HSP70:ASP481:OD2 | Carbon Hydrogen Bond |

**References:**

[1] K. Liang, L. Yang, J. Kang, B. Liu, D. Zhang, L. Wang, W. Wang, Q. Wang, *Asian J Pharm Sci* **2024**, *19*, 100963.
